# Wild boar tuberculosis in Iberian Atlantic Spain: a different picture from Mediterranean habitats

**DOI:** 10.1186/1746-6148-9-176

**Published:** 2013-09-08

**Authors:** Marta Muñoz-Mendoza, Nelson Marreros, Mariana Boadella, Christian Gortázar, Santiago Menéndez, Lucía de Juan, Javier Bezos, Beatriz Romero, María Francisca Copano, Javier Amado, José Luis Sáez, Jorge Mourelo, Ana Balseiro

**Affiliations:** 1 Servicio de Sanidad Animal, Consejería de Medio Rural y de Mar, Xunta de Galicia, Edificio Administrativo San Caetano, Santiago de Compostela, A Coruña, 15781, Spain; 2 SaBio-IREC (CSIC-UCLM-JCCM), Ronda de Toledo s.n., Ciudad Real, 13005, Spain; 3 Departamento de Biología Molecular del Laboratorio de Sanidad y Producción Animal de Galicia, Avenida de Madrid 77, Lugo, 27002, Spain; 4 VISAVET Health Surveillance Centre, Universidad Complutense, Avda, Puerta de Hierro s/n, Madrid, 28040, Spain; 5 Facultad de Veterinaria (Departamento de Sanidad Animal), Universidad Complutense, Avda, Puerta de Hierro s/n, Madrid, 28040, Spain; 6 Laboratorio de Sanidad Animal del Principado de Asturias, Travesía del hospital 96, Gijón, Asturias, 33299, Spain; 7 Dirección General de Sanidad de la Producción Agraria MAGRAMA, Madrid, 24014, Spain; 8 SERIDA, Servicio Regional de Investigación y Desarrollo Agroalimentario, Centro de Biotecnología Animal, Deva-Gijón, Asturias, 33394, Spain

**Keywords:** Wild boar, Tuberculosis, Mycobacterial infections, Atlantic Spain, Cattle

## Abstract

**Background:**

Infections with *Mycobacterium bovis* and closely related members of the *Mycobacterium tuberculosis* complex (MTC) are shared between livestock, wildlife and sporadically human beings. Wildlife reservoirs exist worldwide and can interfere with bovine tuberculosis (TB) eradication efforts. The Eurasian wild boar (*Sus scrofa*) is a MTC maintenance host in Mediterranean Iberia (Spain and Portugal). However, few systematic studies in wild boar have been carried out in Atlantic regions. We describe the prevalence, distribution, pathology and epidemiology of MTC and other mycobacteria from wild boar in Atlantic Spain. A total of 2,067 wild boar were sampled between 2008 and 2012.

**Results:**

The results provide insight into the current status of wild boar as MTC and *Mycobacterium avium* complex (MAC) hosts in temperate regions of continental Europe. The main findings were a low TB prevalence (2.6%), a low proportion of MTC infected wild boar displaying generalized TB lesions (16.7%), and a higher proportion of MAC infections (4.5%). Molecular typing revealed epidemiological links between wild boar and domestic – cattle, sheep and goat – and other wildlife – Eurasian badger (*Meles meles*) and red fox (*Vulpes vulpes*) – hosts.

**Conclusions:**

This study shows that the likelihood of MTC excretion by wild boar in Atlantic habitats is much lower than in Mediterranean areas. However, wild boar provide a good indicator of MTC circulation and, given the current re-emergence of animal TB, similar large-scale surveys would be advisable in other Atlantic regions of continental Europe.

## Background

Bovine tuberculosis (TB) is caused by infection with *Mycobacterium bovis* and closely related members of the *Mycobacterium tuberculosis* complex (MTC) [1]. These infections are shared between livestock, wildlife and sporadically, human beings. This re-emerging zoonotic disease is receiving increased attention from scientists and government agencies. Wildlife reservoirs exist worldwide and can interfere with cattle TB eradication efforts [2,3]. There are three significant wildlife MTC maintenance hosts in Europe, namely the Eurasian badger (*Meles meles*), mainly in the United Kingdom (UK) and Republic of Ireland (RoI), the Eurasian wild boar (*Sus scrofa*), mainly in Mediterranean Spain and Portugal, and deer belonging to the subfamily *Cervinae* such as the red deer (*Cervus elaphus*) in several regions throughout Europe [4].

**Figure 1:**
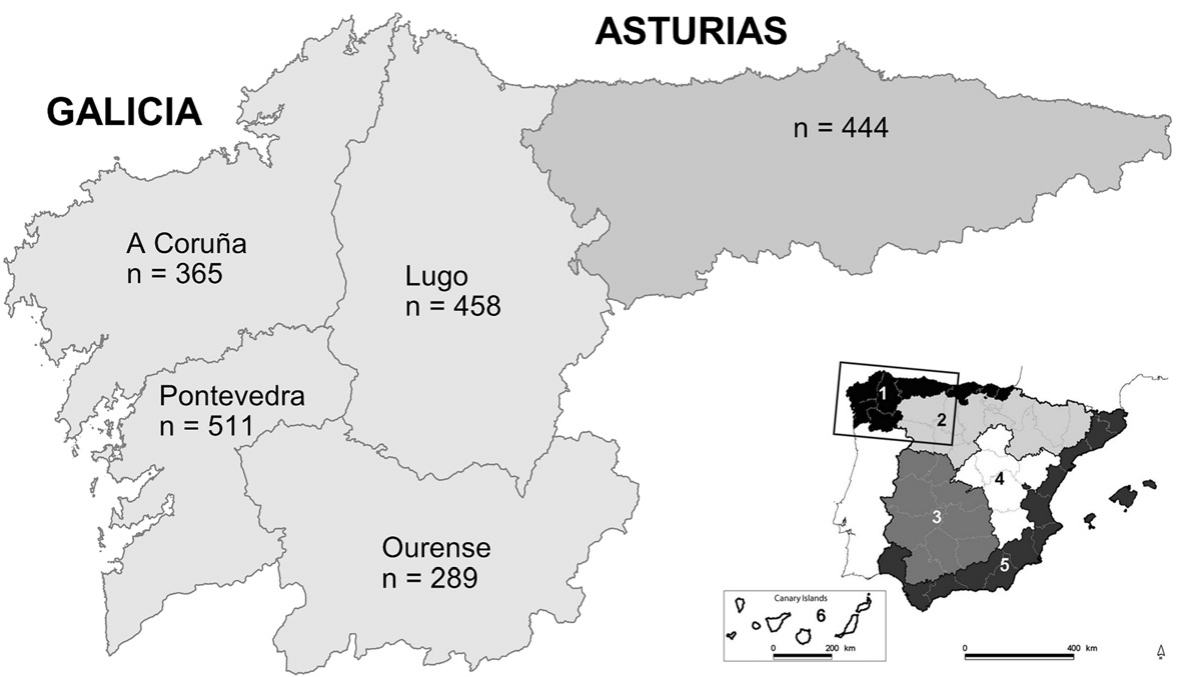
**Bioregions (BR) in Spain and number of wild boar sampled in BR1.** On the right: map of Spain divided into six large BR for sampling and wildlife disease monitoring. Magnified region: number (n) of wild boar sampled in Atlantic Spain (Asturias and Galicia). Both communities belong to the Atlantic BR1 of Spain.

Based on habitat, climate features and wildlife population characteristics, Spain can be divided into six bioregions (BR) [5]; the humid and temperate Atlantic Spain would be represented by BR1, including Galicia and Asturias among other regions, along the north coast of the Iberian Peninsula (Figure 1). Continental Mediterranean habitats would be represented by BR3. In Spain, TB in cattle declined significantly from a herd prevalence of 2.24% in 2002 to 1.33% in 2011 [6]. However, the distribution of positive cattle herds in Spain, based on positive single tuberculin skin test confirmed by culture, is not uniform, with higher prevalence (up to 6%) in Mediterranean habitats of the south and west of the Spanish mainland and low (<1%) prevalence elsewhere (Table 1) [6]. In 2011 Asturias and Galicia reported cattle herd prevalences of 0.14 and 0.19%, respectively. However, both regions included hotspots (Local Veterinary Units) where cattle herd prevalence was >4%.

**Table 1:** **Prevalence of tuberculosis (TB) by single tuberculin skin test and culture in cattle herds in different regions in Mediterranean (BR3, Figure**1**) and Atlantic Spain (BR1, Figure**1**) from 2008 to 2011**[6]

	**BR3, representative of Mediterranean Spain**			**BR1, Atlantic Spain**	
**Year**	**Andalucía (%)**	**Castilla la Mancha (%)**	**Extremadura (%)**	**Galicia (%)**	**Asturias (%)**
2008	5.80	11.62	3.37	0.11	0.22
2009	8.94	10.27	3.78	0.22	0.21
2010	8.54	7.11	3.04	0.28	0.18
2011	6.16	5.35	3.11	0.19	0.14

BR3 is characterized by much higher incidence of TB in cattle herds than BR1.

Wildlife monitoring is needed to identify changes in disease occurrence and to measure the impact of interventions [7,8]. Wildlife Disease Surveillance Schemes have provided the tools for wildlife disease monitoring. These schemes have been in place in Atlantic Spain in Asturias and Galicia since 2001 and 2008, respectively, and have been pivotal for instance for assessing the TB situation of badgers in this region. The recent increase in awareness of TB in wildlife prompted surveillance of badgers in several regions and countries with suitable badger habitats in continental Europe, including Atlantic Spain. Research has estimated prevalence of *M. bovis* infection to be 12.4% in badgers in northern Spain, with spatial correlation of isolates from cattle and badgers [9,10].

One recommendation for monitoring diseases in wildlife is selecting the most appropriate wildlife hosts [8]. Wild boar are considered the best TB surveillance target because of their wide habitat range, high abundance and easy accessibility for sampling as a game species. Moreover, wild boar are easy to test for TB because of their lesion distribution [11,12] and the availability of specific and sensitive antibody tests [13]. MTC infection has been diagnosed in about 50% of wild boar from South-Western Spain [12,14], and in 11.11% of wild boar from Portugal [15]. Outside the Iberian Peninsula, wild boar TB has been reported from eight European countries [4], including one local survey with a prevalence of 42% in Normandy (France) [16], and prevalences of 3% in northern Italy [17], and 3.6% in Switzerland and Liechtenstein [18]. This demonstrates an increasing importance of this ungulate regarding TB in Europe. However, no systematic studies in wild boar have been carried out in Atlantic Spain, although a seroprevalence between 0.01-5% was established in a recent cross-sectional serosurvey [19], confirming the wild boar as a sensitive host to MTC infection.

There are few reports of *Mycobacterium avium* complex (MAC) spp. infections in wild boar. *M. avium* subsp. *hominissuis* was isolated from two wild boar in Portugal [20] and *M. avium* subsp. *avium* from three and four wild boar in Italy and Spain, respectively [21,22]. A recent study has reported a *M. avium* prevalence of 11% by culture in Southern Spain [23]. MAC infections in wild boar are of potential relevance due to the possible diagnostic interference with MTC infections by serology or pathology, particularly if culture and identification by polymerase chain reaction (PCR) is not performed.

The aims of the present study were (1) to document the MTC and MAC situation in wildlife of Atlantic Spain, with a focus on wild boar (considered a good target species) using serological, bacteriological, molecular and pathological diagnostic techniques, (2) to identify associations between infected wild boar populations, infected cattle and other infected domestic and wildlife species, and (3) to establish epidemiological and spatial links between MTC strains from domestic animals and wildlife in a particular region.

## Methods

### Ethics statement

All samples were taken from legally hunted wild boar. Sampling procedures were approved by the Animal Research Ethics Committee of SERIDA (reference numbers 041/06-01-2008 and 010/07-01-2011). We also had the permission to use the samples from the goverment (Sección de Caza de la Consejería de Medio Ambiente del Principado de Asturias and Consejería de Medio Rural y de Mar de la Xunta de Galicia). Animals were hunted and no animals were killed specifically for this study. All data from domestic animals derive from the official testing schemes and did not require animal experiment ethics approval.

### Study area

Free-ranging wild boar from Asturias and Galicia (4.5E to 9.3 W and 41.8 N to 43.8 N) were studied (Figure 1). In these regions, wild boar are harvested through largely non-commercial hunting, and neither large scale fencing nor artificial feeding exists.

Both regions belong to the Atlantic bioregion of Spain (BR1) [5]. BR1 is characterized by an Atlantic climate with a high average precipitation of > 1000 mm/year and mild winter average temperature. Main geographical features are the Atlantic coast to the north and west and the Cantabrian Mountains to the south. Cattle farming (>73,000 herds), both dairy and beef, is a significant activity in both regions. Culture and molecular typing data were available for cattle culled as part of the National Program for the Eradication of TB between 2008 and 2012 in both regions [24], and also for sheep, goat, badger, roe deer (*Capreolus capreolus*) and fox (*Vulpes vulpes*) (Table 2) [10,24].

**Table 2:** **Number of domestic and wildlife species subjected to culture in Galicia and Asturias between 2008–2012 and prevalence of *M. tuberculosis *complex (MTC) and *Mycobacterium avium *complex (MAC) mycobacteria**[6,9,24]

	**Domestic animals**			**Wildlife species**		
	Cattle*	Sheep**	Goat**	**Badger**	Roe deer**	Fox**
**Total cultured**	4,768	167	109	182	350	107
**MTC-infected (%)**	934 (19.59)	82 (49.10)	43 (39.45)	14 (7.69)	0 (0.0)	2 (1.87)
**MAC-infected (%)**	48 (1.01)	0 (0.0)	0 (0.0)	16 (8.79)	4 (1.43)	2 (1.87)

*Cattle subjected to culture were first positive by the single tuberculin skin test during national campaigns of eradication of TB. **Note that sampling of sheep, goat, roe deer and fox was performed mainly in areas with a previous history of TB in cattle.

### Wild boar studied

A total of 2,067 wild boar were sampled between 2008 and 2012 (Table 3): 1,623 originated from Galicia (divided into four provinces) and 444 from Asturias (one province). Thus, the sampled wild boar originated from five provinces (Figure 1). Wild boar were harvested by hunters during the legal hunting season (October to February). The number of sampled wild boar per hunting season was heterogeneous; 2007–2008: n = 6, 2008–2009: n = 163, 2009–2010: n = 300, 2010–2011: n = 643, 2011–2012: n = 916. No data on hunting season was available for 39 wild boar.

**Table 3:** Number of samples from wild boar analyzed and diagnostic technique performed in each region of Atlantic Spain between 2008 and 2012

**Regions BR1**	**Technique**			
	**Culture**	**ELISA**	**Culture/ELISA**	**Culture/histology**
**Galicia**	831	613	265	-
**Asturias**	444	444	-	70
**Total**	1,275	1,057	265	70

In Galicia, official veterinarians in collaboration with hunters’ associations collected blood samples from the heart of the animals, and tissue samples (lungs, tonsils and mandibular, tracheobronchial, mediastinal and mesenteric lymph nodes-LN) were taken, along with sex and age data of each wild boar for further processing. In Asturias, a trained veterinarian sampled blood and tissues (including also retropharyngeal LN) in the field immediately after hunting. The sex ratio was almost balanced with 954 females and 930 males. No data on sex was available for 183 wild boar. Age was determined according to the tooth eruption pattern. There were n = 220: <12 months, n = 688: < 30 month and n = 289 animals older than 30 months. Age data was not available for 870 wild boar.

### Bacteriological studies

The Mycobacteria Growth Indicator Tube (MGIT) liquid medium system, Löwenstein-Jensen solid media with sodium pyruvate and Coletsos solid media [25,26] were used to isolate members of the MTC and *M. avium* complex (MAC) in samples from 1,275 wild boar sampled from areas with history and/or high prevalence of TB in cattle (Table 3). Specific media to isolate *M. avium* subsp. *paratuberculosis* were not used in this study. Pools of tissues (2 g) from the lungs, tonsils and mandibular, tracheobronchial, mediastinal and mesenteric LN (in Galicia) or from the lungs and the retropharyngeal, mandibular, tracheobronchial and mediastinal LN (in Asturias) of each wild boar were decontaminated using the BBL MycoPrep Becton Dickinson kit (BD Diagnostic Systems, USA). MGIT liquid medium was incubated at 37°C for at least 6 weeks using the automated BACTEC MGIT 960 (BD Diagnostic Systems, USA). Solid media were incubated at 37°C for at least 10 weeks.

### Identification and molecular characterization of isolates

Real Time-Polymerase Chain Reaction (qPCR) to identify MTC species was performed on culture isolates using the MTC forward primer 5’-TAGTGCATGCACCGAATTAGAACGT-3’, the MTC reverse primer 5’-CGAGTAGGTCATGGCTCCTCC-3’ and the TaqMan probe YY/BHQ 5’-AATCGCGTCGCCGGGAGC-3’, which amplifies a 184 base pair fragment [27]. RT-PCR amplification involved an initial denaturation cycle at 95°C for 10 min, followed by 40 cycles of denaturation, annealing and extension at 95°C for 30 s and 60°C for 1 min.

MTC isolates were characterized by DVR-spoligotyping [28] following hybridisation of biotin-labelled PCR products onto a spoligotyping membrane (Isogen Bioscience BV and VISAVET ‘home-made’ membrane). Results were recorded in SB code followed by a field of four digits according to the *M. bovis* Spoligotype Database website [29]. The MTC isolates were compared with those identified in cattle and badger (when available) from the same area [10,24]. To confirm the similarity of isolates from cattle, wild boar and other domestic and wildlife species from the same geographical location, Mycobacterial Interspersed Repetitive Units - Variable Number Tandem Repeats (MIRU-VNTR) typing was performed [30] using four (ETR-A, ETR-B, QUB11a, QUB3232) or nine (ETR-A, ETR-B, ETR-D, ETR-E, MIRU26, QUB11a, QUB11b, QUB26 and QUB3232) VNTR markers.

Identification of MAC species was performed on positive cultures [31], using the MAC forward primer 5’-AACGTGTTCTACCTCTGCGGGGCAAG-3’ (nucleotides 34–59 in the sequence GenBank X68102), the MAC reverse primer 5’-CCGGGAGAGTAGGTCATGGCTCC-3’ (nucleotides 259–281 in the sequence GenBank X68102) and the TaqMac Cy5/BHQ 5’-CGCTCGGCACTAAAAGGCAGTGG-3’ (nucleotides 221–240 in the sequence GenBank X68102) (Cultek, Spain), which amplifies a 248 bp fragment. RT-PCR was carried out at 95°C for 10 min, followed by 40 cycles of 95°C for 30 s and 60°C for 1 min. PCR amplification of IS*1245*, IS*901* and IS*901*-flanking region (FR300) genomic targets was carried out as MAC sub-species can be readily differentiated on the basis of their IS*1245*, IS*901* and FR300 genotype. Thus, *M. avium* subsp. *avium* is IS*1245*+, IS*901*+ and FR300− (a band at 1742 bp is present, but there is no band at 300 bp), while *M. avium* subsp. *hominissuis* is IS*1245*+, IS*901*− and FR300+. *M. avium* subsp. *silvaticum* is IS*1245*+, IS*901*+ and FR300+, and *M. intracellulare* is negative for all three targets.

### Serological study

A recently developed enzyme-linked immunosorbent assay (ELISA) was used to identify seropositive wild boar [13]. This test uses a bovine tuberculin purified protein derivative (bPPD) as coating antigen and protein G horseradish peroxidase as conjugate. The test has a moderate sensitivity (79.2%) and good specificity (100%) in detecting MTC infected wild boar. Sera from 1,057 wild boar were tested by ELISA (Table 3), following the protocol described previously [13].

### Gross and histopathological examinations

The TB lesion pattern has been well described previously in wild boar from Mediterranean Spain [11,32]. Macroscopic TB lesions in Mediterranean wild boar were classified as “localized”, if only one anatomical region was affected, or “generalized”, if at least two different anatomical regions were affected, and were termed “A” if lesions were less than 10 mm in diameter and “B” if they were over 10 mm diameter. Microscopic lesions were also classified as “granuloma 1”, initial granulomas composed of mixed inflammatory cells only or with a necrotic core; “granuloma 2”, slightly necrotic-calcified granulomas with scarce calcium deposits in the necrotic core; and “granuloma 3”, strongly necrotic-calcified granulomas with a calcification area similar to or bigger than the necrosis area [11]. We used the same classification for wild boar from Atlantic Spain.

Gross lesions observed in 70 wild boar from Asturias were carefully recorded based on Martín-Hernando et al. [11]. Mandibular, retropharyngeal, mediastinal, and tracheobronchial LN (up to 0.5 cm thickness), and lung and spleen (up to 2 cm thickness) were sliced for further macroscopic observation. Any other organ showing lesions suggestive of TB was processed in the same way as lungs.

Samples for histopathology were taken from the mandibular, retropharyngeal, tracheobronchial and mediastinal LN, lungs and spleen of 70 Asturian wild boar (Table 3). In one case liver was also collected because of gross lesions. They were fixed in 10% neutral buffered formalin and processed routinely. Several sections of 4 μm were cut from each sample and stained with haematoxylin and eosin (HE) and by Ziehl-Neelsen (ZN) method for acid-fast bacteria (AFB). In selected positive cases showing TB-like lesions immunohistochemical examination by means of the peroxidase anti-peroxidase (PAP) method was performed [33]. The sections were incubated with specific anti-*M. bovis* serum (Dako, Denmark), diluted 1 in 2,000. To evaluate the specificity of the anti-*M. bovis* antibody, tissue samples from a tuberculous cow were used. Normal non-infected rabbit serum (Dako, Denmark) was used as a negative control.

### Statistical analysis

Differences in prevalence between years, sexes, age classes, were assessed with the two tailed Fisher’s exact test (FET) for both ELISA and culture results. In case of multiple comparisons, the Bonferroni correction was applied. Significance level was set at P < 0.05. Agreement between serology and culture was assessed by Cohen’s Kappa coefficient using 265 wild boar which were analyzed by both techniques (Table 3). Statistical analysis of wild boar data was performed with the R statistic software (version 2.14.2) [34] in combination with the “vcd” package (version 1.2-12) [35] for calculation of the Cohen’s Kappa coefficient.

### Spatial cluster analysis

Wild boar were identified using the Cartesian coordinates of the centroid of their hunting area of origin. Presence of geographic clusters of TB was assessed with the SaTScan Software (version 9.1.1, http://www.satscan.org). For this purpose, the Bernoulli model for purely spatial clusters was chosen, which was conducted in an iterative way. Geographic data on hunting areas was obtained from the Consejería de Medio Rural y de Mar de la Xunta de Galicia, and the Consejería de Medio Ambiente del Principado de Asturias. Geographic data visualization and processing was performed with the GvSIG software (version 1.12, http://www.gvsig.org).

## Results

### Culture, identification of isolates and spoligotyping

**Figure 2:**
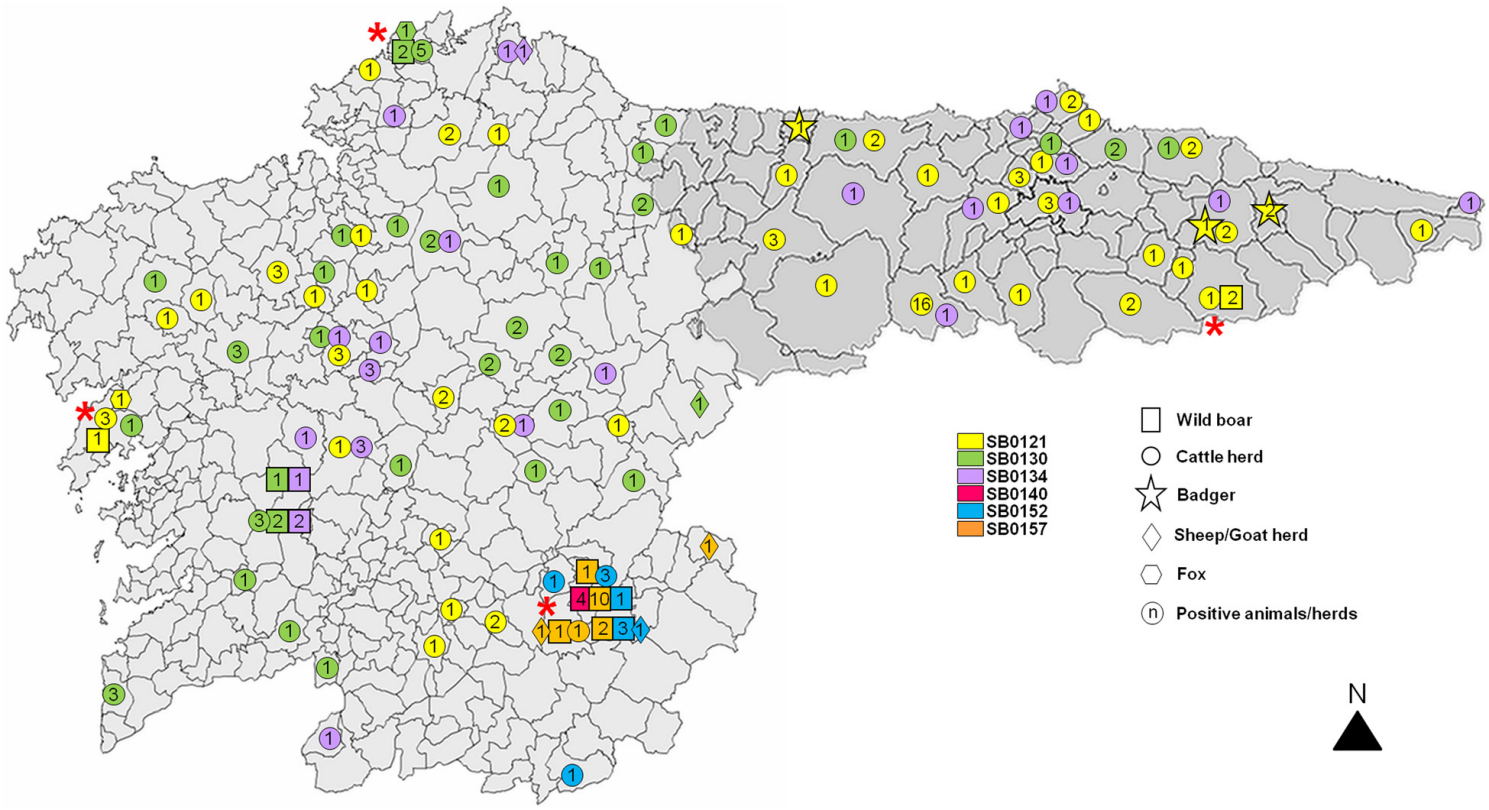
**Tuberculosis-positive domestic and wildlife species in Atlantic Spain.** Shared *Mycobacterium bovis* spoligotypes identified in wild boar and other species from 2008 to 2012 are shown. Red asterisks indicate that isolates from domestic and wildlife species share spoligotype profiles in the same geographical areas.

**Figure 3:**
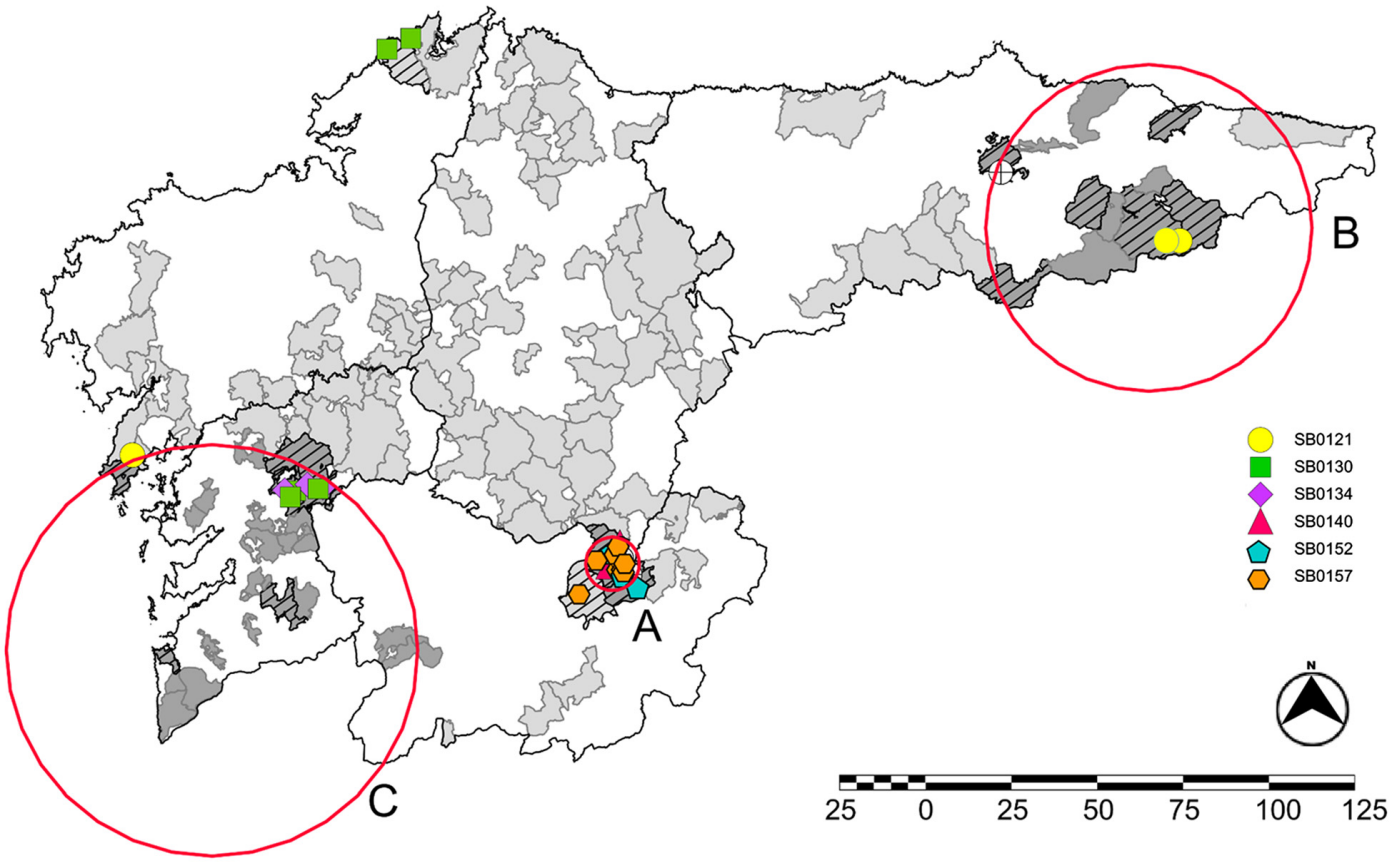
**Tuberculosis-positive wild boar in Atlantic Spain and the *Mycobacterium bovis *spoligotypes identified.** Shaded areas represent hunting areas where wild boars were sampled. Striped areas represent hunting areas where at least one wild boar was found positive for TB. Dark shaded areas represent hunting areas included in the cluster with higher risk of TB. Different forms represent the spoligotypes identified. Spatial cluster analysis revealed 3 significant clusters **(A**, **B** and **C**; relative risk 7.87, 5.98, 10.71; P < 0.001, 0.019, 0.013, respectively**)**.

MTC species were isolated and identified by RT-PCR from 33/1,275 wild boar (2.59%; 95% CI: 1.8-3.6, Table 4). No significant difference was found between sex, age class or hunting season. The isolates were identified as *M. bovis* (n = 19) or *M. caprae* (n = 14) and were characterised by spoligotyping as SB0157*-M. caprae* (n = 14), SB0130*-M. bovis* (n = 5), SB0152*-M. bovis* (n = 4), SB0140*-M. bovis* (n = 4), SB0134*-M. bovis* (n = 3) and SB0121*-M. bovis* (n = 3) (Figures 2 and 3). MIRU-VNTR analysis of the four loci (ETR-A, ETR-B, QUB11a, and QUB3232) subdivided the isolates typed as SB0157 in 5 MIRU-VNTR types, SB0130 in 3 VNTR types, and SB0140 in 2 VNTR types (Table 5). One SB0121 isolate from wild boar could not be typed by MIRU-VNTR. In order to track the dissemination of the disease we selected at least one isolate from cattle farms (n = 30) with the same spoligotyping profile and from the same area and isolates from other animal species [badger (n = 3), sheep (n = 1), goat (n = 3) and fox (n = 1)] and they were also analysed by VNTR typing.

**Table 4:** **Prevalence of *M. tuberculosis *complex (MTC) and *M. avium *complex (MAC) species infection, in wild boar in Atlantic Spain, using culture, serology and pathology**

	**Culture of MTC**	**Culture of MAC**	**ELISA *M. bovis***	**Pathology *M. bovis***			
				**Gross lesions**	**Generalized lesions**	**Localized lesions**	Microscopic lesions and IHC* for *M. bovis*
**Wild boar**	33/1,275	58/1,275	22/1,057	6/70	1/6	5/6	9/70
	(2.59%)	(4.55%)	(2.08%)	(8.57%)	(16.7%)	(83.43%)	(12.86%)

* *IHC* Immunohistochemistry was performed on animals with microscopic tuberculosis-like lesions.

**Table 5:** **Identification and genotyping of the *Mycobacterium tuberculosis *complex isolates (n = 33) from wild boar in Atlantic Spain**

**Identification**	**Spoligotyping profile (N isolates)**	MIRU-VNTR types (N isolates)^**^
*M. caprae*	SB0157 (14)	5-3-6-8 (9)
		4-3-6-8 (2)
		5-3-6-7 (1)
		1-3-6-8 (1)
		6-3-10-10 (1)
*M. bovis*	SB0121 (3^*^)	4-4-10-8 (2)
	SB0130 (5)	4-5-10-9 (1)
		4-5-10-10 (2)
		4-5-9-8 (2)
	SB0134 (3)	3-5-10-5 (3)
	SB0140 (4)	6-3-10-10 (2)
		6-3-F-F (1)
		4-5-6-8 (1)
	SB0152 (4)	7-5-8-7 (4)

^*^ DNA from an SB0121 isolate was not available.

^**^ The order of the MIRU-VNTR loci is ETR-A, ETR-B, QUB11a, and QUB3232. Bold MIRU-VNTR profiles are the most frequent profiles. F. Failure of amplification.

Relationships between the *M. bovis/M. caprae* isolates from wild boar and the other animal species are shown in Figure 2. The strain SB0157-*M. caprae* was the most frequently identified in wild boar (14/33, 42.42%) and was only identified once in cattle (1/627, 0.16%; odd ratio = 441.5, FET: P < 0.001, Figure 2). The 4 or 9-loci MIRU-VNTR profile of the wild boar, fox and cattle isolates within the spoligotype SB0121 was the same and minor differences at one locus (MIRU26, QUB11a, or QUB11b) were associated to the SB0130, SB0134, and SB0157 profiles, respectively, although they were considered as belonging to the same clonal complex. However different MIRU-VNTR profiles were found in wild boar and cattle with the SB0152 profile. Similar MIRU-VNTR profiles were associated to wild boar and two caprine flocks with SB0157 profile. Spoligotype SB0140*-M. bovis* found in wild boar was not identified in cattle or in other species during the study period. VNTR profiling of SB0121 revealed that the strains identified in badgers (5-4-3-3-5-F-2-5-8) and cattle (4-4-3-3-5-10-2-5-8) were different but closely related.

MAC species were isolated from 58/1,275 wild boar (4.55%; 95% CI: 3.5-5.8). PCR analysis of the isolates revealed *M. avium* subsp. *avium* (n = 15) and *M. avium* subsp. *hominissuis* (n = 43).

### Serological results

Twenty-two out of 1,057 wild boar were positive by ELISA (2.08%; 95% CI: 1.3-3.1, Table 4). No significant differences were found between sex, age class or hunting season.

### Comparison between culture and ELISA

Cohen’s Kappa coefficient of agreement between MTC positive culture and ELISA was 0.71. We therefore considered the agreement as fairly good and thus considered pooling the samples for general statistics as appropriate.

### Overall statistic and spatial cluster analysis

Forty-six wild boar were positive by culture or ELISA (2.22%; 95% CI: 1.6- 3.0). Spatial cluster analysis revealed 3 significant clusters (A, B and C; relative risk 7.87, 5.98, 10.71; P < 0.001, 0.019, 0.013, respectively; Figure 3). Three spoligotypes were found in cluster A (SB0157-*M. caprae*, SB0152-*M. bovis* and SB0140-*M. bovis*), which were only found in this cluster (Figure 3). Three other spoligotypes were found in cluster C, one of which was only found in this cluster (SB0134*-M. bovis*). SB0121-*M. bovis* was the only spoligotype found in cluster B.

### Pathological investigations

**Figure 4:**
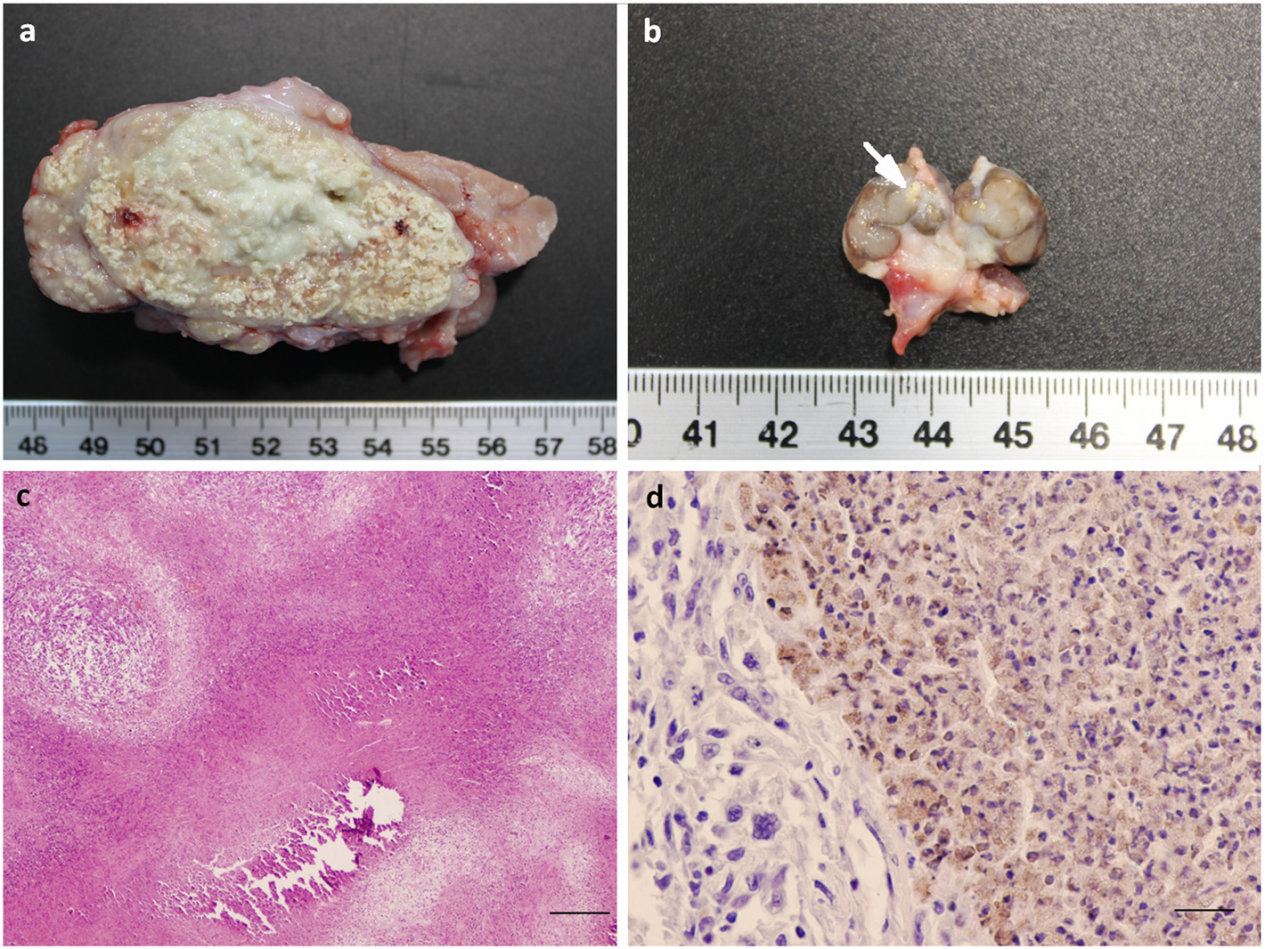
**Tuberculous lesions in wild boar from Asturias. a)** Severely enlarged mandibular lymph node from a wild boar showing generalized tuberculosis. **b)** Typical lesion found in mandibular lymph node in wild boar in northern Spain, consisting of small area of calcified necrosis (arrow) confined to one anatomical region. **c)** Histological section of a mandibular lymph node. Granuloma “type 2” consisted of necrotic-calcified granulomas, with few calcium deposits in the necrotic core. Haematoxylin-eosin staining, bar = 200 μm. **d)** Liver. Positive immunostaining for *Mycobacterium bovis* located in macrophages in the necrotic area. PAP staining, bar = 20 μm.

Gross TB-like lesions were observed in 6 wild boar (6/70, 8.57%; 95% CI: 3.2-17.7) from Asturias (Table 4), 5 showing “A” (small) lesions (5/6, 83.3%) and only one “B” (large) lesions (1/6, 16.7%) (Figure 4a). Five animals had lesions confined to one anatomical region; four of them in the mandibular LN (Figure 4b) and one in the liver. Only one animal (the one with “B” lesions) presented generalized TB-lesions located in the head, thorax and abdomen. SB0121-*M. bovis* was isolated from this wild boar.

Histopathology allowed us to confirm the presence of TB lesions in a total of 9 (9/70, 12.85%) wild boar from Asturias, three of them with no visible gross lesions but with typical features of bovine tuberculous granulomas in lungs. Granulomas were classified as Type 1 in three wild boar, Type 2 in three and Type 3 in another three wild boar (Figure 4c). MTC was confirmed by immunohistochemistry. A multifocal intense positive immunolabel was observed in the necrotic areas and in macrophages within and around the granulomas (Figure 4d). AFB were demonstrated with ZN staining in 5/9 cases. Two of these wild boar were positive by culture.

Wild boar infected with MAC showed histopathological lesions indistinguishable from those caused by MTC. However, immunohistochemistry carried out on these animals using the anti-*M. bovis* antibody was negative. Additionally 9 (9/70, 12.85%) wild boar showing gross lesions initially compatible with TB in the mandibular LN, were diagnosed as having actinomycosis based on histopathological morphology.

## Discussion

This is the first cross-sectional survey of wild boar TB in Atlantic habitats combining culture, pathology and serology. The results provide insight into the current status of wild boar as MTC hosts in temperate regions of continental Europe. The main findings were a low TB prevalence, a low proportion of MTC infected wild boar displaying generalized TB lesions, and a higher proportion of MAC infections.

This study provides much needed information on the TB status of wild boar in Atlantic Spain, confirming the presence of mycobacterial infections in these animal populations. The prevalence of MTC infections based on culture (2.59%) and serology (2.08%) was low. This is in agreement with the low prevalence of seropositive wild boar recorded in Atlantic habitats in a previous nation-wide survey (<5%) [19]. In contrast, a 52% MTC infection prevalence using culture was recorded in Doñana National Park in south-western Spain [2], and high infection prevalences have repeatedly been recorded in other regions of Mediterranean Spain and Portugal [4,36]. The prevalence of antibodies against MTC was estimated at 35% in Mediterranean Spain (BR3) [19]. This large difference between the high prevalence in wild boar from Mediterranean regions and the low prevalence in those from Atlantic ones might be due to differences in the presence of several, not mutually exclusive risk factors for MTC infection in wildlife. First, in Atlantic Spain, the prevalence of TB in cattle herds is very low contrary to the high prevalence of TB in cattle in Mediterranean Spain (Table 1) [6]. Second, wild boar occur at higher densities in Mediterranean habitats than in Atlantic ones in Spain [37,38]. Third, artificial management of wild boar for hunting purposes, including fencing and feeding, is much more common in Mediterranean habitats [14,39]. Fourth, dry habitats in Mediterranean Spain facilitate aggregation of animals when watering or feeding (especially during the summer), enabling the transmission of the disease. Such spatial aggregation and intra- or inter-specific contacts at waterholes or feeders are less likely to occur in humid Atlantic habitats.

The proportion of lesions found in Atlantic Spain was markedly lower than in Mediterranean habitats. There may be different explanations for the low proportion of individuals with generalized disease observed in the present study (16.7%). The small sample size available for pathological study could be one of these reasons. This proportion contrasts with the much higher proportion found in Mediterranean Spain (57.8%) [11]. Interestingly, a recent study has demonstrated how the proportion of severe/generalized cases of TB among wild boar increases in dry years in southern Spain, although infection prevalence did not increase [Vicente J, unpublished observations]. This was probably a result of higher food restriction and TB susceptibility, or of augmented aggregation at waterholes. In our study, the prevalence of confirmed TB gross lesions was also low (8.57%) as compared to Mediterranean Spain (82.68%). Lesions were mainly restricted to small size ones in a single organ -the mandibular LN. Thus, it seems that the likelihood of MTC excretion by wild boar in Atlantic Spain is much lower than in Mediterranean habitats, and that most of the infected animals would shed small amounts of bacteria, if any.

Prevalence of histopathological lesions in Asturias was higher (9/70, 12.85%) than prevalence obtained by using culture (2/70, 2.86%). The histopathological findings suggest that an exhaustive pathological study of tissues increases prevalence and that we may have missed infected wild boar by only using culture. The gold standard for TB diagnosis is post-mortem examination with bacteriological confirmation [2]. The number of tissues examined and their pooling may have impaired the sensitivity of culture in this study. However, the average small size of the lesions found in Atlantic Spain has also to be taken into account since it may impair the isolation of mycobacteria. Sampling additional tissues would have increased the sensitivity, as shown for badgers [40].

In this study ELISA results showed a good agreement with culture and this assay has been a pivotal tool for the diagnosis and cost-effective large scale monitoring of TB in this area. Wild boar blood can be easily and cleanly collected [41], and ELISA has proven to be a useful first screening tool. Moreover, ELISA can also be used for detecting areas at risk for targeted surveys based on more sensitive techniques. If this approach is proposed in other Atlantic areas where wild boar TB is understudied, the bigger picture of the epidemiology in the wild boar TB could be revealed.

Wildlife and cattle in the same areas frequently share the same MTC strains [10,14,42]. The most frequent spoligotype found in cattle and badger in Spain was SB0121-*M. bovis*[10]. However, in this study the most frequent spoligotype in wild boar was SB0157-*M. caprae* (42.42%) and SB0121 was the least frequent. We identified six different *M. bovis*/*M. caprae* spoligotypes, four of which (SB0121*-M. bovis*, SB0130*-M. bovis*, SB0134*-M. bovis* and SB0157*-M. caprae*) had previously been identified in cattle, two (SB0152*-M. bovis* and SB0157*-M. caprae*) in goat and sheep and two (SB0121*-M. bovis* and SB0130*-M. bovis*) in fox. VNTR analysis contributed with additional information about the strains and demonstrated that cattle isolates with the SB0121, SB0130, SB0134, and SB0157 profiles were geographically associated with wild boar, suggesting an epidemiological link between these species. By contrast VNTR study of the isolates from the wild animal species and cattle with the SB0152-*M. bovis* did not find any link. The finding of a strain pattern in wild boar (SB0140*-M. bovis*) that was neither detected in cattle nor in other species during the study period would suggest some cases of independent MTC circulation among wild boar. This was not seen in badgers in previous studies [10]. Also, the identification of SB0157-*M. caprae* strain in wild boar (42.42%) more frequently than in cattle (0.16%) suggests some differences between the wild and domestic cycles of MTC epidemiology in this study area. However, it must be taken into account that SB0157-*M. caprae* was identified in one single “hot-spot” area where cattle, goats and sheep also shared this strain.

MAC species (*M. avium* subsp. *avium* and *M. avium* subsp. *hominissuis*) were identified in wild boar at similar prevalences than MTC species, as also happened with sympatric badgers [9]. This indicates circulation of these bacteria in the region in both species. Interestingly, these mycobacteria have not been identified in cattle in Atlantic Spain in such prevalences (see Table 2). In Mediterranean Spain, wild boar infection by MAC species is always lower than MTC [21,23,43]. Since these infections cause gross and histological lesions undistinguishable from TB, MAC infections must be considered when diagnosing MTC in wild boar from Atlantic habitats. As demonstrated in this study, conventional histology supplemented by immunohistochemistry to enhance specificity, would help to detect MTC in wild boar in future studies.

MTC is a multi-host pathogen modulated by host behaviour, abundance, exposure and susceptibility [4]. Whilst in Mediterranean Iberia this multi-host system is based on cattle, wild boar and red deer; in Atlantic Spain the multi-host system would include larger numbers of domestic animals (cattle, sheep and goat) and wildlife species (mainly badger and wild boar), although at lower TB prevalences. Risk factors for TB maintenance in wild boar like aggregation and high densities are not present in Atlantic Spain; but badgers are also infected in this region and could contribute to a ‘mixed-species reservoir’ *sensu*[44]; nevertheless, overall the existence of a wildlife reservoir such as in Mediterranean areas seems unlikely.

When TB lesions are observed in infected badgers, these occur often in the lungs (suggesting oro-nasal and fecal excretion) and kidneys (suggesting excretion by urine) [40]. Regarding wild boar, studies in high-prevalence regions have shown high proportions of generalized TB with lung involvement, strongly suggesting oro-nasal and fecal shedding [11]. In Atlantic Spain, 0.83% and 0% infected badgers showed visible lung and kidney lesions, respectively [9]. In this study 1.43% wild boar showed visible lung lesions, therefore suggesting a higher risk of transmission.

In any case, the presence of wildlife (wild boar and badger) contributes to the risk associated with cattle TB in farms in northern Spain [Cowie CE, unpublished observations], thus the risk of infection spillback from this species to cattle cannot be excluded. As for badgers, the risk of transmission to cattle might include direct contact on pasture and in buildings, and contamination of pasture, water and cattle feed [45]. Therefore, cautionary measures to prevent contact between cattle and wild boar, and gralloch removal after hunting should be carried out. Also, wild boar population monitoring as well as wild boar TB surveillance should be continued and improved.

## Conclusions

This study confirms that (a) wild boar carry MTC mycobacteria at a low prevalence in Atlantic habitats in northern Spain, probably because of climate, management and because of the main reservoir, cattle, also have a low TB prevalence, (b) wild boar from this study area present less generalized lesions than in Mediterranean Spain, possibly due to lower TB prevalence in cattle, different management, habitat and climate conditions, (c) typing reveals similar profiles to those detected in other domestic – cattle, sheep and goat – and wildlife – badger and fox – hosts in this region, suggesting epidemiological links between these species and (d) our results are relevant in the current scenario of increasing wild boar densities [37] and sympatric wildlife TB hosts in Atlantic Spain [10] and elsewhere in continental Europe [18,46]. To summarize, the wild boar is currently regarded as a spillover TB host in Atlantic Spain, although its status may change based on local or regional risk factors, such as an increase of cattle TB, wild boar abundance, land use changes and changes in cattle and wildlife management practices [14].

## Competing interests

None of the authors of this paper has a financial or personal relationship with other people or organisations that could inappropriately influence or bias the content of the paper.

## Authors’ contributions

MMM, AB, NM, CG: contributed to the conception, design, and data collection, laboratory work, drafting and writing of the manuscript. LDJ, JB, BR, MB, MFC, JA, SM, JM, JLS: contributed to laboratory work, data analysis and drafting of the manuscript. All authors have read and approved the final manuscript.
